# Genome Sequence of the Basidiomycete White-Rot Fungus* Trametes pubescens* FBCC735

**DOI:** 10.1128/genomeA.01643-16

**Published:** 2017-02-23

**Authors:** Zoraide Granchi, Mao Peng, Thomas Chi-A-Woeng, Ronald P. de Vries, Kristiina Hildén, Miia R. Mäkelä

**Affiliations:** a GenomeScan B.V., Leiden, The Netherlands; b Fungal Physiology, CBS-KNAW Fungal Biodiversity Centre and Fungal Molecular Physiology, Utrecht University, Utrecht, The Netherlands; c Division of Microbiology and Biotechnology, Department of Food and Environmental Sciences, University of Helsinki, Helsinki, Finland

## Abstract

Here, we report the genome sequence of the basidiomycete white-rot fungus *Trametes pubescens* FBCC735, isolated from Finland. The 39.67-Mb genome containing 14,451 gene models is typical among saprobic wood-rotting species.

## GENOME ANNOUNCEMENT

Wood-rotting basidiomycete white-rot fungi are predominantly responsible for natural mineralization of the aromatic lignin polymer, and *Trametes* spp. are known as efficient lignin degraders (1). *Trametes pubescens* is a hardwood-degrading saprobe, commonly found in Europe and in Northern America, which can produce a diverse set of lignin-modifying enzymes (2).

The *T. pubescens* FBCC735 dikaryon (HAMBI-Fungal Biotechnology Culture Collection, University of Helsinki, e-mail: fbcc@helsinki.fi) was maintained on 2% (wt/vol) malt extract 2% (wt/vol) agar (MEA) plates. Four mycelium-covered plugs (Ø 7 mm) from MEA plates were used to inoculate 100 mL 2% (wt/vol) malt extract liquid cultures that were incubated stationary at 25°C for 21 days. Genomic DNA was extracted using cetyltrimethylammonium bromide–based buffer (3). For RNA extraction, the fungus was cultivated on solid-state cultures that contained 2 g (dry weight) of 2.5-cm Norway spruce (*Picea abies*) wood sticks or 2-cm wheat (*Triticum aestivum*) straw pieces on 1% (wt/vol) water agar at 25°C for 21 days. Moisture content of the cultures was adjusted to 60% with sterile H_2_O. The cultures were inoculated with 4 mL of homogenized *T. pubescens* mycelium (4) from low-nitrogen asparagine medium, pH 4.5 (5) containing 1% (vol/vol) glycerol, and incubated stationary at 25°C for 21 days. RNA was extracted using CsCl ultracentrifugation (6) and checked using a Fragment Analyser (Advanced Analytical Technologies). DNA concentration was determined using Qubit (Life Technology), while gDNA quality was verified using a 0.6% agarose gel.

gDNA was fragmented using a Focused-ultrasonicator (Covaris). The NEBNext Ultra DNA library prep kit and NEBNext Ultra Directional RNA library prep kit for Illumina (E7370S/L and E7420S/L, respectively) were used for library preparation. Lab-on-a-Chip analysis and Fragment Analyzer were used for quality and yield checks of the library with fragments of approximately 300 to 500 bp and 500 to 800 bp.

Illumina cBot and HiSeq 2500 standard Illumina primers and HiSeq control software HCS version 2.2.58 were used for clustering and DNA sequencing with concentrations of 8.0 pM of DNA and 16.0 pM of cDNA. The Illumina data analysis pipeline RTA version 1.18.64 and Bcl2fastq version 1.8.4 were used for image analysis, base calling, and quality checking.

FASTQFilter version 2.05, a GenomeScan in-house pipeline, was used for adapter removing and quality checking: bases with Phred scores above Q22 and reads longer than 36 bp passed the filtering.

For the assembly, Abyss version 1.3.7 (7) with a *k*-mer length of 64 was used. Scaffolds shorter than 500 bp were removed. A total of 2,173 contigs was used for the assembly of the 39.67-Mb genome. The GC content was 57.50% as assessed by QUAST (8). The *T. versicolor* genome was used as a gene-finding trainer for the HMM-based algorithm Glimmer (version 3.02) (9). Mapped mRNA-Seq reads were used by the CodingQuarry (10) software tool for an evidence-based method of gene finding. Combining the two methods, gene models for 14,451 genes were obtained.

### Accession number(s).

This whole-genome shotgun project has been deposited in DDBJ/ENA/GenBank under accession number MNAD00000000. The version described in this paper is the first version and is also available through MycoCosm (11).
